# Optimizing pediatric asthma education using virtual platforms during the COVID-19 pandemic

**DOI:** 10.1186/s13223-022-00713-y

**Published:** 2022-08-07

**Authors:** Dhenuka Radhakrishnan, Andrea Higginson, Madhura Thipse, Marc Tessier, Arun Radhakrishnan

**Affiliations:** 1 Children’s Hospital of Eastern Ontario, 401 Smyth Road, Ottawa, ON K1H 8L1 Canada; 2 Children’s Hospital of Eastern Ontario Research Institute, Ottawa, Canada; 3 Department of Pediatrics, University of Ottawa, Ottawa, Canada; 4 ICES, Ottawa, ON Canada; 5 Department of Community and Family Medicine, University of Toronto, Toronto, Canada

**Keywords:** Telemedicine, Asthma, Pediatrics, Pandemic, Education

## Abstract

**Background:**

We compared patient and caregiver knowledge and confidence for managing asthma, and participant experiences when comprehensive asthma education was delivered in person versus in the virtual setting.

**Methods:**

We performed a multi-methods study using structured surveys and qualitative interviews to solicit feedback from patients and caregivers following participation in a comprehensive asthma education session between April 2018 and October 2021. We compared participant knowledge and confidence for managing asthma as well as user experience when the education was attended in-person or virtually. Quantitative responses were summarized descriptively, and qualitative feedback was analyzed for major themes.

**Results:**

Of 100 caregivers/patients who completed post education satisfaction surveys and interviews, 52 attended in person and 48 virtually, with the mean age of patients being 6.7 years (range: 1.2–17.0). Participant reported gains in knowledge and confidence for asthma management were not different between groups and 65.2% preferred attending virtual asthma education. The majority of participants described virtual education as a safer modality that was more convenient and accessible.

**Conclusions:**

We demonstrated the successful implementation of a novel, virtual asthma education program for patients and caregivers of children with asthma. Both virtual and in-person delivered asthma education were equally effective for improving perceived knowledge and confidence for asthma self-management and virtual education was considered safer, more convenient and accessible. Virtual asthma education offers an attractive and effective option for improving the reach of quality asthma education programs and may allow more children/patients to benefit.

**Supplementary Information:**

The online version contains supplementary material available at 10.1186/s13223-022-00713-y.

## Background

Asthma is the most common pediatric chronic disease with a prevalence in Canada of 15–25% [1]. Typical symptoms include wheeze, cough and dyspnea that when poorly controlled lead to impaired quality of life and recurrent acute care visits. Many cases of poor asthma control relate to lack of knowledge around self-management of the disease [2–4]. For this reason asthma education is considered the cornerstone of management and is emphasized in Canadian and international asthma guidelines [5, 6]. Asthma self-management education is shown to improve asthma medication adherence and health outcomes, including symptoms, exacerbations, and school/work absenteeism, but several factors limit its widespread delivery. The most obvious limitation relates to lack of resources to support a sufficient number of knowledgeable asthma educators to serve the population. Group asthma education delivered in classroom settings is effective and can simultaneously reach a larger number of patients and families. However, many centres lack physical space to support large groups. Furthermore, infection control issues that are highlighted during the COVID-19 pandemic limit the utilization of group asthma education. Another issue is that families often do not return for their education appointment following hospital discharge. Providing education during an acute care visit to the hospital, is attractive in terms of capitalizing on a captive audience, but is often unsuccessful due to the anxiety and fatigue of parents who are actively supporting a sick child [7]. Virtual asthma education can overcome many of these barriers, but has not been well studied for its efficacy or family/patient acceptance. In this study, we compare patient and caregiver knowledge and confidence for managing asthma, and their experiences in attending comprehensive asthma education when delivered in person or in the virtual setting.

## Methods

The Children’s Hospital of Eastern Ontario (CHEO) is a tertiary care pediatric facility in Ottawa, Ontario and serves a population of 2 million people. Comprehensive asthma education is provided as a standard of care for families of children and adolescents following hospitalization for an asthma exacerbation, or following a new diagnosis of asthma. Education is provided by certified asthma educators, and covers content including an explanation of the pathophysiology of asthma, signs and symptoms, criteria for diagnosis, trigger avoidance, asthma medication and adherence and development of a personalized asthma action plan. Asthma education is provided individually at the bedside, as small group classroom sessions (2–3 patients/families) or as a separate visit within 4 weeks of discharge. Outpatients are provided education during their clinic visit. However, since March 2020, due to the COVID-19 pandemic, most patients receive education virtually, via the PHIPA compliant web-conferencing platform, Zoom [8].

Since April 2018, we conducted monthly semi-structured telephone interviews with a random sample of up to 5 caregivers/patients who attended comprehensive asthma education sessions to solicit their feedback. The interviews consisted of 7 scripted and closed-ended survey questions; 4 that ascertained patient/parental perception of their knowledge gained in various domains of asthma management, and 3 that measured confidence in managing different aspects of their/their child’s asthma, with responses recorded on a 5-point Likert scale (See Additional file 1: Tables S1–S3). Participants were posed 5 additional open-ended questions to provide a better understanding of their responses to the survey, and to ascertain their experience with the format and duration of the session, the understandability and importance of the content, the demeanor of the educator and perceived benefits. Any negative or constructive feedback was also solicited. Participants were asked about their experience in navigating the video-conferencing platform and their comfort and preference with receiving asthma education in the virtual or traditional in-person setting. All responses were tape-recorded and transcribed for analysis.

We collected and descriptively analyzed information on basic demographics (e.g., age of patient, caregiver gender) using proportions, means and ranges. Responses to scripted interview questions were descriptively summarized by calculating and comparing the proportion of participants with a given response, and responses to open ended questions were analyzed qualitatively for content and themes. Qualitative analysis was conducted independently by two individuals, followed by a discussion and agreement on the organization and presentation of major themes.

## Results

Between April 2018 and October 2021, a Total of 362 comprehensive asthma education sessions were provided to children following an asthma hospitalization or new outpatient asthma diagnosis; 104 education sessions were virtual. A total of 100 caregivers/patients completed the post education satisfaction interviews, and of these, 52 attended the session in person and 48 participated virtually. All families who were approached to participate in the post education interviews agreed (100% response rate).

Overall, the mean age of the asthma patient population was 6.7 years (range: 1.2–17.0 years), with a younger mean age and range noted amongst children whose caregivers attended in-person education prior to the COVID-19 pandemic (Table 1). Among the survey respondents, 75 were female primary caregivers, 21 were male primary caregivers, and 4 were adolescent patients (age 14–17 years old). A higher proportion of female caregivers participated in the education session virtually, compared to in-person.

**Table 1:** Characteristics of study sample and overall impression of asthma education

Characteristic	In-person, pre pandemic (n = 29)	In-person, during pandemic (n = 23)	Virtual, during pandemic (n = 48)
Mean patient age years, (range)	4.1 (1.2–11.0)	9.5 (1.7–17.0)	6.6 (1.4–17.0)
Female caregiver n, (%)	19 (65.5)	17 (73.9)	39 (81.3)
Adolescent participant n, (%)	0	1	3
Preferred n, (%)			
Virtual		6 (12.5)	41 (85.4)
No preference	N/A	13 (27.0)	5 (10.4)
In-person		4 (17.4)	2 (4.2)
Rating of education session n, (%)			
Very good	28 (96.6)	21 (91.3)	46 (95.8)
Good	1 (3.4)	2 (8.7)	2 (4.2)

Overall, amongst the post-pandemic participants, the majority (65.2%) expressed that they preferred the option of a virtual session over an in-person session, 13.1% preferred in-person, and 21.7% did not have a preference. No patients/caregivers had difficulty connecting to the virtual session, and 95% of patients/caregivers who attended either in-person or virtual education rated their experience as “very good”, while 100% “strongly agreed” that other families should attend.

**Fig. 1:**
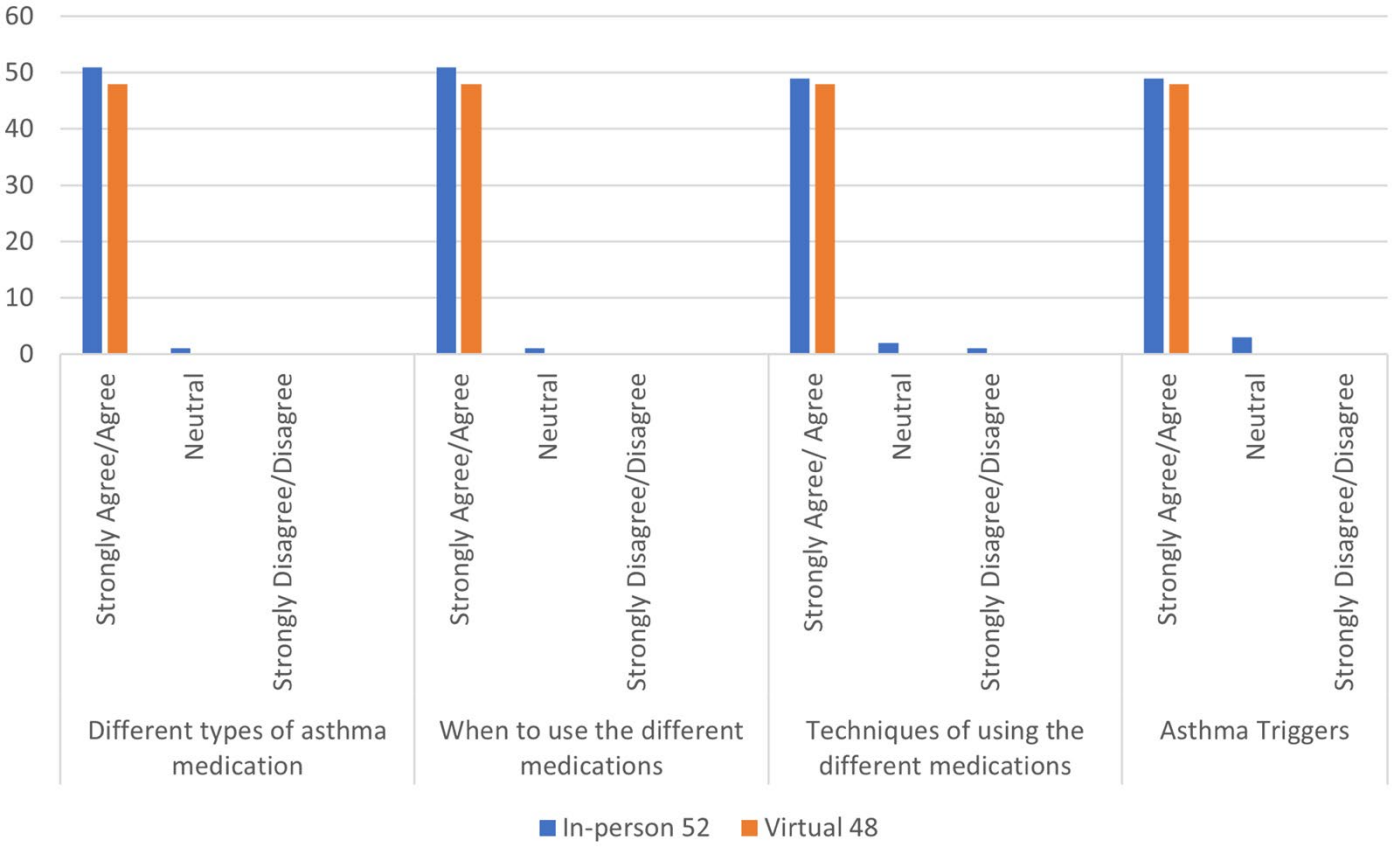
Impact of asthma education on patient/caregiver perceived asthma management knowledge

**Fig. 2:**
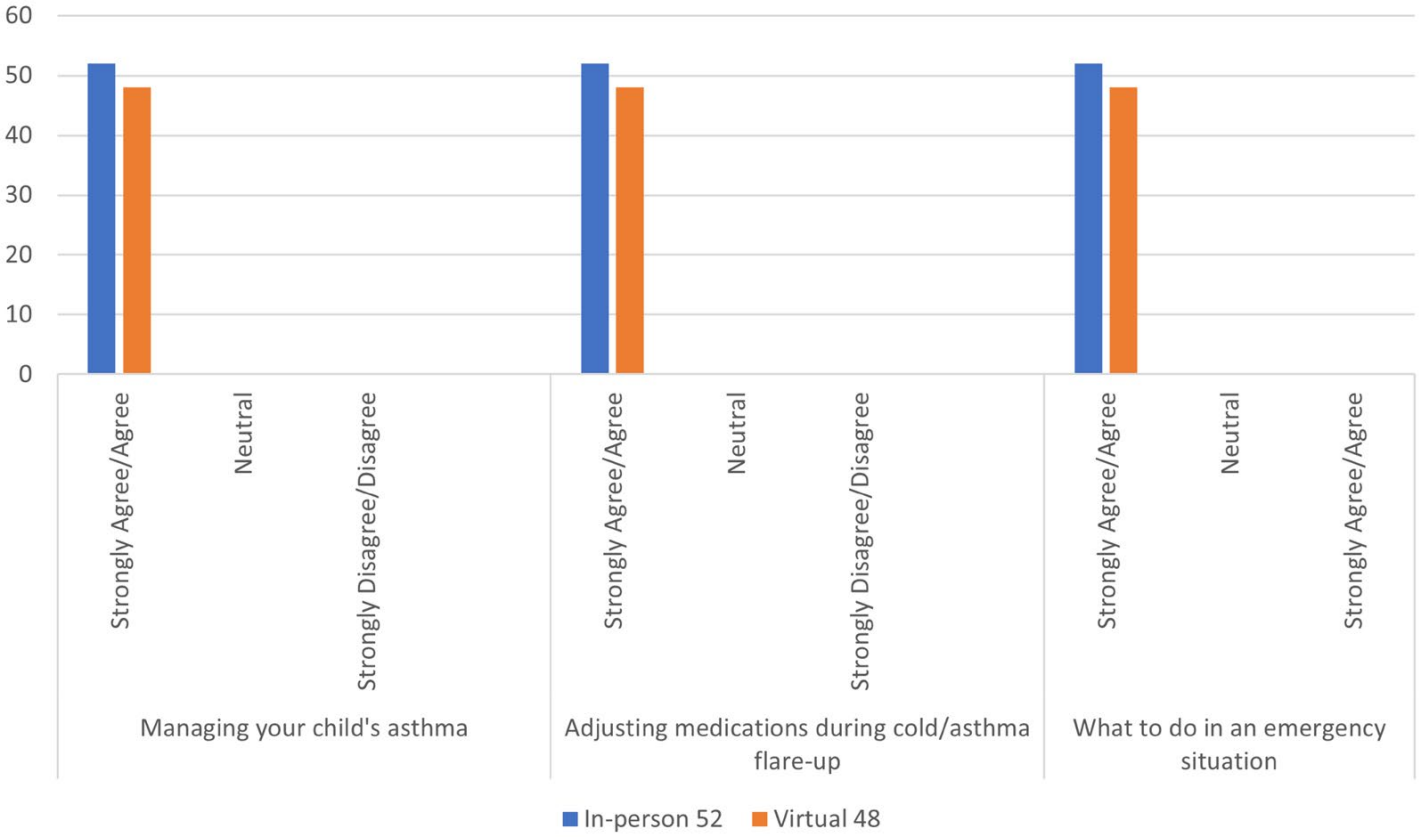
Impact of asthma education on patient/caregiver confidence in disease self-management

Regardless of education setting, participants agreed that they had improved knowledge in understanding their/their child’s different types of asthma medications (99%), when to use them (99%), the correct technique for administering asthma medications (98%) and their/their child’s asthma triggers (97%), with no significant difference between groups. (Fig. 1) All participants (100%) agreed that they had improved confidence in managing their/their child’s asthma, adjusting medications during an asthma flare-up and what to do during an emergency situation. (Fig. 2).

### Qualitative analysis—major themes

Participants of both in-person and virtual education had predominantly positive comments to share, with their education encounter described in relation to three main themes*: information, value and experience*. (Table 2). Participants stated the education sessions provided comprehensive and detailed *information* that was simple and easy to understand. Overall, participants felt the education sessions were *valuable* for improving their understanding of asthma, and for providing them with management strategies to prevent future exacerbations. A small number of participants who attended group sessions (both in-person or virtual) stated a preference for a more personalized experience; in contrast some caregivers appreciated being able to share a common experience with other participants in the group. Some participants stated they wished they had received the education earlier and expressed gratitude for the opportunity to attend.

**Table 2:** Participant feedback on asthma education

Theme		Responses	
1. Information (detailed, understandable)		“I learned quite a bit about asthma” “The session was very informative. The educator explained the concepts in simple language which was easy…to understand” “Not until we had the information did we realize that there was so much that we didn’t know, and that there was so much that we were doing incorrectly with our other child”	
2. Value (knowledge impact, felt supported)		“The education session taught us how to manage my child's asthma so that we don’t have to go to the hospital frequently” “Wish we had received it [education] years ago” “Over the moon that we were able to have the education otherwise we wouldn’t know what to do.” “A lot more confidence about when to give things, when to go to the hospital. Now we have a plan that is straight forward.” “Session was exactly what we needed”	
		**Attended in-person**	**Attended virtually**
3. Experience	Safety	“We needed a bigger room…so many people in the little room”	“I want to protect my child from any virus.” “I am not comfortable with social media.” “I am concerned about privacy and confidentiality.”
	Convenience	“It would be beneficial to have education during the [hospital] stay rather than coming in after the fact.” “If possible—offering weekend sessions—possibly an evening could work for us as well.”	“I have another young child and sometimes it is difficult to manage the schedule and come to CHEO in person.” “Saves [commuting] time” “We do not have to commute and worry about parking. “Virtual session suit[s] our lifestyle better.”
	Accessibility	“Both the parents should be allowed to attend the session if interested.”	“My husband and child both could attend the session since it was over Zoom” “Since we live in the countryside our internet connection is not very stable. It was not difficult to connect to the session but due to poor internet service there was a lag or other technical issues.”

A third theme related to the *experience* of participants and was divided into the sub-themes of *safety*, *convenience*, and *accessibility*, with both positive and negative experiences expressed. All participants commented positively about the educators who were described as patient, helpful, professional, knowledgeable, friendly and supportive. A small proportion of in-person attendees felt uncomfortable to use social media, and shared concerns about data privacy; however, the overwhelming majority of those who attended virtual education, and some who attended in-person believed the virtual setting to be safer for avoiding infection. Among those who attended in-person, many commented on the inconvenience of having to return to the hospital for education, as well as some frustration that the sessions were limited to just one caregiver due to lack of large classroom space. Among the majority of patients/caregivers who expressed a preference for virtual education, most cited it to be more convenient, and accessible in terms of time- and cost-savings and flexible to their lifestyle. Participants in virtual education appreciated being able to include additional family members to participate, and not having to organize childcare to attend a session at the hospital. In contrast, some who preferred to attend in-person sessions shared difficulties in trying to access the virtual technology, due to slow home internet connections.

## Discussion

In this single-center multi-methods study, we demonstrated the successful implementation of a novel, virtual asthma education program for patients and caregivers of children with a recent asthma hospitalization or newly diagnosed asthma. Asthma education was effective for improving perceived knowledge and confidence for asthma self-management. Virtual asthma education was equally effective for achieving these outcomes as in-person education. Participants described their experience in asthma education according to three main themes: information, value, and experience. We did not identify significant differences between groups with respect to the first two themes, but in-person versus virtual education attendees had different experiences, specifically related to sub-themes of safety, convenience, and accessibility.

The demonstration of improved knowledge and confidence among caregivers in our study is in keeping with the prior literature on telemedicine in asthma. While most previous studies focus on the outcomes of remote clinical care, a small number do focus on education interventions alone. A recent study in adult patients with obstructive lung disease, including asthma, demonstrated the efficacy of an asynchronous patient-initiated virtual video education intervention for teaching correct inhaler technique [9]. Only a limited number of prior studies have examined synchronous video telemedicine compared to in-person comprehensive asthma education, with most conducted as school-based interventions among lower income children. A systematic review that included 5 studies conducted in children 3–17 years old demonstrated inconsistent and inconclusive effects of virtual asthma education on clinical outcomes, asthma knowledge and satisfaction compared to usual care. However, there was some evidence of improved quality of life, symptom management ability and symptom burden in the intervention groups, indicating that virtual asthma education is likely at least as beneficial as in-person education interventions [10, 11].

Experiences of participants of virtual or in-person education differed in relation to safety, convenience, and accessibility. The majority of patients and families who participated in virtual asthma education (and some who attended in-person during the pandemic) preferred the virtual option, for the reason of improved safety and infection exposure avoidance. From a provider perspective, infection prevention was the reason for our program’s reliance on this modality since the COVID-19 pandemic. Our study results indicate our virtual education program allowed uninterrupted delivery of high-quality comprehensive asthma education for eligible patients despite reductions to in-person interactions for safety and infection control reasons. However, a small minority of caregivers expressed safety and privacy concerns with the use of social media (i.e. Zoom) and preferred an in-person education experience.

Beyond infection avoidance, there were additional unanticipated benefits of virtual asthma education, including convenience for families. Participants expressed their preference in receiving asthma education from the comfort of home, thereby avoiding the inconveniences of travel time and cost, and organizing care for other children while attending an appointment at the hospital. These findings were similar to a small (n = 30) pre-pandemic study in which 84% of caregivers of preschoolers with asthma who participated in a single synchronous 1-hour telemedicine asthma education session found it convenient and useful, though emergency room visits and hospitalizations were not impacted [12].

Another obstacle overcome by virtual care was accessibility, which is traditionally limited by lack of space and educators. While small classroom sizes limit the number of attendees for in-person education sessions, many participants expressed the desire for additional family members to attend. In contrast, the virtual space is unlimited and caregivers appreciated the ability for multiple family members to attend. Small classroom sizes pose an infection control/safety risk, even pre-pandemic, and have restricted the access of the virally-triggered hospitalized adolescent patient and symptomatic caregivers to attend group education. However, there is limited capacity for one on one bedside education and many caregivers find it difficult to concentrate and absorb bedside teaching while also tending to their sick child [7]. Providing adequate supervision of preschool patients while their parent attends a classroom education session is an ongoing challenge, and further limits access to education during the hospital stay. Conversely, virtual education, scheduled after hospital discharge, alleviated the issue of organizing childcare and optimized attendance. High cost and time for travel for in-person clinical care are additional recognized barriers in access to care, and in Canada, have been addressed through the (infrequent) use of specialized telemedicine sites and government travel grants (restricted to those living  > 200 km from a care facility) [13]. However, the inconveniences of travel for those living closer to the hospital continue to limit in-person attendance.

A few barriers in access to virtual education identified by participants included the requirement for reliable high-speed internet access which was an impediment for some families [14] and detracted from the quality of the education experience. To mitigate this, and avoid inequity related to the ‘digital divide’ [15] it would be important to continue to offer in-person education for certain families; those without adequate internet access, and those who may not prioritize attendance after hospital discharge for financial and other psychosocial reasons.

Limitations of the current study include the lack of true randomization and the likelihood that many patients who engaged in virtual education may have been biased towards a positive response to this modality given the current pandemic situation. Whether positive uptake will continue months to years after COVID-19 related physical distancing restrictions have eased and there is resumption of previous levels of in-person medical care is unclear. Our study was also conducted during a period when asthma symptom burden was generally reduced due to the reduction in viral respiratory triggers compared to previous years [16] and this may have influenced results. For this reason, we were unable to compare health outcomes such as symptom burden, quality of life and exacerbation frequency among patients who receive virtual versus in-person education though this could be studied in future.

Despite some limitations, the results of this study suggest a potential for harnessing virtual care delivery to broaden the reach of our asthma education program to include a larger number of patients, including those in the community. Wide acceptance of virtual education and studies in adults demonstrating the efficacy of group asthma education [10] support increasing the size of group virtual sessions, participation, and impact. However, larger groups may lead to less personalization. Determining a threshold maximum number of participants per virtual session beyond which the quality of the teaching diminishes is an additional area for future study.

A shift to virtual clinical care in a variety of medical settings across North America, and the receptiveness of patients and families provided a unique opportunity to establish a virtual asthma education program at our tertiary care pediatric center. This program was well received by participants, as it provides a safe and infection-free setting for asthma education. We anticipate that this virtual education program will have continued good uptake given its multiple additional benefits, including convenience, and accessibility, without compromise on quality. However, allowing families the option of in-person or virtual education remains an important consideration as some families continue to prefer care in-person, or may have limited digital access. For the care provider, virtual asthma education has the potential to enhance and broaden the reach of our tertiary care asthma program and in future, allow us to offer high quality, expertly delivered asthma education to a broader group of patients, including those living in rural and remote communities.

## Supplementary Information

**Additional file 1: Table S1.** Asthma education session feedback for in-person and virtual participants. **Table S2.** Impact of asthma education on parent/caregiver knowledge about the disease. **Table S3.** Impact of asthma education on parent/caregiver confidence of disease self-management. **Figure S1.** Impact of asthma education on participant knowledge and confidence in asthma self-management following attendance at an in-person or virtual comprehensive asthma education session.

## Data Availability

Additional data available upon request.

## Abbreviation

PHIPA: Personal Health Information Protection Act
