# Supplementary material for: Optimizing pediatric asthma education using virtual platforms during the COVID-19 pandemic
Source: Allergy Asthma Clin Immunol. 2022 Aug 7;18:72. doi: 10.1186/s13223-022-00713-y (PMC9358084; doi:10.1186/s13223-022-00713-y)
Supplement: Supplementary file 1 — Additional file 1: Table S1. Asthma education session feedback for in-person and virtual participants. Table S2. Impact of asthma education on parent/caregiver knowledge about the disease. Table S3. Impact of asthma education on parent/caregiver confidence of disease self-management. Figure S1. Impact of asthma education on participant knowledge and confidence in asthma self-management following attendance at an in-person or virtual comprehensive asthma education session. [file 13223_2022_713_MOESM1_ESM.docx]

**Supplementary Files**

Table S1. Asthma education session feedback for in-person and virtual participants

| **Category** | **Item*** | **Response** | **Number of Responses**  **(total N = 100)** |
| --- | --- | --- | --- |
| **Knowledge about** | Different types of asthma medication | Agree | 99 |
|  |  | Neutral | 1 |
|  |  | Disagree | 0 |
|  | When to use the different medications | Agree | 99 |
|  |  | Neutral | 1 |
|  |  | Disagree | 0 |
|  | Techniques of using the different medications | Agree | 97 |
|  |  | Neutral | 2 |
|  |  | Disagree | 1 |
|  | Asthma Triggers | Agree | 97 |
|  |  | Neutral | 3 |
|  |  | Disagree | 0 |
| **Confidence about** | Managing your child's asthma | Agree | 100 |
|  |  | Neutral | 0 |
|  |  | Disagree | 0 |
|  | Adjusting medications during cold/asthma flare-up | Agree | 100 |
|  |  | Neutral | 0 |
|  |  | Disagree | 0 |
|  | What to do in an emergency situation | Agree | 100 |
|  |  | Neutral | 0 |
|  |  | Disagree | 0 |

**Participants were asked to rate their knowledge or confidence about each of the listed items following an asthma education session, according to the following Likert Scale: Strongly Agree, Agree, neither agree nor disagree, Disagree or Strongly Disagree. Responses of Strongly Agree and Agree were grouped together and similarly, responses of Disagree or Strongly Disagree were grouped together.*

**Table S2. Impact of asthma education on parent/caregiver knowledge about the disease**

| **Item*** | **Response** | **In person**  **n (%)** | **Virtual**  **n (%)** |
| --- | --- | --- | --- |
| Different types of asthma medication | Agree | 51 (98.07) | 48 (100) |
|  | Neutral | 1 (1.92) | 0 |
|  | Disagree | 0 | 0 |
| When to use the different medications | Agree | 51 (98.07) | 48 (100) |
|  | Neutral | 1 (1.92) | 0 |
|  | Disagree | 0 | 0 |
| Techniques of using the different medications | Agree | 49 (94.23) | 48 (100) |
|  | Neutral | 2 (3.84) | 0 |
|  | Disagree | 1 (1.92) | 0 |
| Asthma Triggers | Agree | 49 (94.23) | 48 (100) |
|  | Neutral | 3 (5.76) | 0 |
|  | Disagree | 0 | 0 |

**Participants were asked to rate their knowledge about each of the listed items following an asthma education session, according to the following Likert Scale: Strongly Agree, Agree, neither agree nor disagree, Disagree or Strongly Disagree. Responses of Strongly Agree and Agree were grouped together and similarly, responses of Disagree or Strongly Disagree were grouped together.*

**Table S3. Impact of asthma education on parent/caregiver confidence of disease self-management**

| ***Item** | **Response** | **In person**  **n (%)** | **Virtual**  **n (%)** |
| --- | --- | --- | --- |
| Managing your child's asthma | Agree | 52 (100) | 48 (100) |
|  | Neutral | 0 | 0 |
|  | Disagree | 0 | 0 |
| Adjusting medications during cold/asthma flare-up | Agree | 52 (100) | 48 (100) |
|  | Neutral | 0 | 0 |
|  | Disagree | 0 | 0 |
| What to do in an emergency situation | Agree | 52 (100) | 48 (100) |
|  | Neutral | 0 | 0 |
|  | Disagree | 0 | 0 |

**Participants were asked to rate their confidence about each of the listed items following an asthma education session, according to the following Likert Scale: Strongly Agree, Agree, neither agree nor disagree, Disagree or Strongly Disagree. Responses of Strongly Agree and Agree were grouped together and similarly, responses of Disagree or Strongly Disagree were grouped together.*

Figure S1. Impact of asthma education on participant knowledge and confidence in asthma self-management following attendance at an in-person or virtual comprehensive asthma education session.
